# Two Pleas for Mrs. Besant

**Published:** 1891-11-07

**Authors:** 


### THEOSOPHY: TWO PLEAS FOR MRS. BESANT.

(By an Anonymous Contributor.)

The natural capabilities for credulity exist in extremely diverse proportions in different individuals. Because one may be unable to appreciate Mrs. Besant's expansive faculties in that direction, it is no reason for pooh-poohing the creed of which she is the exponent.

Instinctive antipathy is one thing, logical scepticism is another. The amusing analysis of theosophy as given in The Hospital of last week, conveys nothing to an unbiased mind; to state simply that 94.5 per cent. of Besant-Olcottian Theosophy is pure twaddle, is illogical, to say the least of it, unless one can also prove the statement; this the writer carefully evades doing by seeking to throw discredit on three of Mrs. Besant's statements.

In the first place, Mrs. Besant is not the foundress of Theosophy, the late Madame H. P. Blavatsky having initiated it into Europe; and that "Asiatic Mysticism" and "pure twaddle" were not necessary constituents of her creed is proved by an autograph letter of hers to Mr. Stead (editor of *Pall Mall Gazette*) in which she terms him a "True Theosophist." Mr. Stead not happening to be a woman (which in Mrs. Besant's case seems to be looked upon as a fault) and being moreover a thoroughgoing, practical man of this 19th Century, and withal endowed with large intellectual capacities, few persons, would have the temerity to accuse him of being the deluded victim of a vivid imagination.

Again, is it not rather absurd to presume that a man of such unquestioned intellect as Erasmus, was not qualified to testify his conviction as to the reality of that which had been proved to him by ocular demonstration? We shall be told next that no one except an experienced astronomer could give a thorough and reliable account of an eclipse.

The arguments that are applied to mesmerism apply equally to hypnotism; either hypnotism is of use as a remedial agent, or it is not; its vaunted cures are real or supposed. Which?—remains for M. Charcot and others to prove. Theosophy as propounded by Madame Blavatsky advocates far more real, practical religion than is practised by half of the goody-goody, namby-pamby, Sunday-go-to-meeting churchgoers of the present day, who only go for the sake of appearances, and who gabble prayers to a Deity whose existence they deny by their deeds, if not by thought or word; and who are either too much wrapped up in conceit or self-satisfaction, or else too culpably lazy to think out the pros and cons of their respective religious (?) tenets and so accept in blind unreasoning faith the creeds and dogmas that were handed down by their fathers.

Surely, instead of casting ridicule and actual condemnation on Mrs. Besant and her theories, we should rather sympathise with the struggles and aspirations of the soul of a member of the great family of God after light. From secularism to theosophy is one important step in the right direction, may the next one land Mrs. Besant out of the darkness of speculative reasoning into the knowledge of the reality of the kingdom of Christ!

---

(By B. A. Cantab.)

Sir,—If the recipe you give in your issue for October 24th represents the conception you have formed of Theosophy, it does not do credit to your intellectual capacity, nor is there any reason why you should put your distorted idea of Theosophy before your readers as Theosophy itself. Please to remember that much of what you doctors talk is "pure twaddle" to us laymen, until we have taken the trouble to master your theories and phraseology. It must require considerable intellectual complacence to imagine that, while Mrs. Besant and her many able and intelligent co-believers are deluded by a system containing 94½ per cent. of "pure twaddle," you doctors remain sane. There are more aspects of truth than that which appeals to the medical understanding, and invaluable though the latter may be in its services to mankind, we must remember that all are not fitted by nature to study truth on its practical side, or from a physiological standpoint. As a doctor, whose aspirations and capacities are fully satisfied by his professional duties, you may have no need of Theosophy, and would, therefore, do well to let it alone, provided you do your duty to your neighbour; but others have their sphere of action in the world of religious and social controversy, and things which appear trivial and unimportant to you, are serious matters for them, and they may be driven to study truth in higher aspects than the merely practical and physiological. The "common-sense" point of view may be very useful, but it is not everything. As to the experiments of Charcot, and those of Mesmer, although there may be difference of *theory* between the two operators, I think no one will fail to recognise the general similarity between Hypnotism and Mesmerism, and to see that the former is a revival of the latter.

